# The Impact of Environmental Fluctuations on Evolutionary Fitness Functions

**DOI:** 10.1038/srep15211

**Published:** 2015-10-19

**Authors:** Anna Melbinger, Massimo Vergassola

**Affiliations:** 1 University of California, San Diego, Department of Physics, 9500 Gilman Drive, La Jolla, CA 92093, USA

## Abstract

The concept of fitness as a measure for a species’ success in natural selection is central to the theory of evolution. We here investigate how reproduction rates which are not constant but vary in response to environmental fluctuations, influence a species’ prosperity and thereby its fitness. Interestingly, we find that not only larger growth rates but also reduced sensitivities to environmental changes substantially increase the fitness. Thereby, depending on the noise level of the environment, it might be an evolutionary successful strategy to minimize this sensitivity rather than to optimize the reproduction speed. Also for neutral evolution, where species with exactly the same properties compete, variability in the growth rates plays a crucial role. The time for one species to fixate is strongly reduced in the presence of environmental noise. Hence, environmental fluctuations constitute a possible explanation for effective population sizes inferred from genetic data that often are much smaller than the census population size.

Since its formulation by Darwin and Wallace, the theory of evolution and its explanation for the ongoing development of different species became a paradigm of modern biology^1^,^2^. Herbert Spencer’s famous expression “survival of the fittest”^3^ provides an appealing and concise summary of the concept of natural selection. However, it leaves aside one of the most complex yet important aspects of evolutionary theory, namely identifying the factors determining the fitness of a species^4^,^5^: fittest individuals are by definition the ones which prevail but the reasons facilitating their survival are not obvious. Even leaving aside the difficulties arising due to the genotype-phenotype mapping^6^, it is far from trivial to identify a species’ fitness function and its dependence on measurable ecological quantities.

Examples of determinants for evolutionary fitness are reproduction-related quantities like birth rate, viability, number of offspring and span of fertility. All of those directly influence the amount of genes that an individual will transmit to the future population (either carried by the individual itself or its offspring). Importantly, all those factors depend on the specific environment in a species’ habitat. This fact is strongly related to the concept of niches: in each niche a different species has potentially the largest fitness and outcompetes less adapted ones. Other ecological factors like population structure and composition also have bearing on this issue. Therefore, traditional fitness concepts solely based on growth rate and viability were extended by frequency-dependent^7^,^8^ or inclusive fitness approaches^9^.

Environmental conditions are not constant but vary on almost every time scale and pattern. Whole niches change with time and space^10^ but also within a well-defined niche constant environmental conditions seem to be the exception rather than the norm. For instance, the availability of different nutrients, the presence of detrimental substances or other external factors like temperature, all strongly influence reproduction/survival and occur on a broad range of time scales^11^. The relevance of environmental fluctuations for evolutionary dynamics was demonstrated in many different contexts, e.g. general consequences of environmental noise on growth and extinction^12^,^13^,^14^,^15^,^16^,^17^,^18^,^19^,^20^,^21^,^22^,^23^,^24^, more specific scenarios like the influence of environmental noise on evolutionary game theory or predator-prey models^25^,^26^, the invasion dynamics of new species^27^, its interplay with phenotypic variations^28^, phenotypic plascitity^29^ or role environmental tolerance^30^. Evolutionary strategies to actively cope with variable environmental conditions like phenotypic heterogeneity or bet-hedging have been extensively studied as well^31^,^32^,^33^,^34^,^35^,^36^.

The scope of this paper is to quantitatively understand the impact of fluctuating reproduction rates on evolutionary dynamics. Specifically, the interplay of such dynamics with demographic fluctuations was not fully elucidated yet. The latter becomes especially important as the crossover between selection driven and fluctuation driven evolution is a major focus of modern research on evolutionary dynamics^37^,^38^,^39^,^40^. Environmental fluctuations potentially influence both neutral and selection driven evolution rendering a proper understanding essential to grasp the dynamics. Here, we investigate this issue by combining analytical calculations and stochastic simulations. Thereby, we show that an individual’s sensitivity to environmental changes contributes substantially to its fitness: a reduced sensitivity increases the fitness and may compensate for large disadvantages in the average reproduction rate. We also find that fluctuating environments influence neutral evolution where they can cause much quicker fixation times than naïvely expected. As we explain in detail in the following, this finding has interesting consequences for the interpretation of the effective population size which is typical characteristic to quantify randomness in an evolutionary process. Finally, we show that our results hold not only for very quickly fluctuating environment but also for switching rates up to the time scale of reproduction.

## Results

To understand the impact of variable environmental conditions, we first consider an extension of a model introduced by May^18^, which is an evolutionary process based on fluctuating birth rates. Different species are defined by their specific traits which influence both their average reproduction rate as well as their sensitivity on environmental changes.The model assumes that populations grow logistically, i.e. the population grows exponentially if the total population size is small but reaches a finite maximal size after a while which is set by limited resources. In contrast to standard logistic or Verhulst dynamics we here consider growth rates which are not necessarily constant but may fluctuate due to environmental changes. Mathematically such a scenario can be modeled by decoupled birth-death dynamics for each trait, *S*, with noise stemming from environmental changes and demographic fluctuations (the latter were not considered in ref. 18). The dynamics is described by the following stochastic differential equations for the total number of individuals, *N*_*S*_, of type *S*:


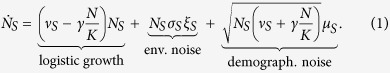


The first term is purely deterministic and accounts for reproduction and death events according to standard logistic growth^41^,^42^. In more detail, an individual of type *S* reproduces at a growth rate, *ν*_*S*_ while the death rates are assumed to be identical for all traits. Population growth is bounded and deaths rates increase with the total population size 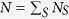 where *N*_*S*_ is the number of *S*-type individuals. This may account for density-dependent ecological factors such as limited resources or metabolic waste products accumulating at high population sizes. For specificity, we choose 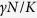 as functional form where *K* is the carrying capacity scaling the maximal number of individuals and 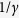 sets the timescale of death.

**Figure 1:**
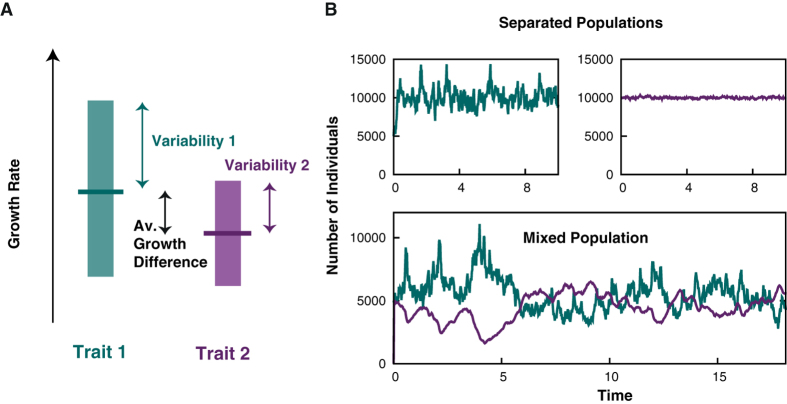
(**A**) Illustration of two species with different growth rates and sensitivities to the environmental fluctuations. The mean growth rates are indicated by horizontal lines and the variabilities by the length of the colored bars. (**B**) Typical trajectories for two species with the same growth rates 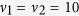 but different sensitivities to the environment 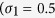 and 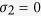, as defined in the text), shown in green and violet, respectively. In the upper two panels the time evolution of two separate non-competing populations is shown. Due to their equivalent average growth rate, both reach the same carrying capacity but fluctuations are substantially stronger for species **1**. In the lower panel the evolution of both species in the same habitat is shown. Other parameters defined in the main text are the death rate 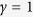 and the carrying capacity *K* = 1000.

Let us now consider the role of environmental noise. We assume that the environment directly acts on the reproduction rates as illustrated in Fig. 1A. Thereby, variable environmental conditions can be modeled by fluctuating birth rates:


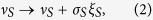


where 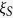 is *δ*-correlated white noise with 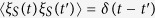 and the standard deviation (STD) 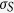 is the strength of that noise. Note that 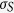 is affected both by the actual noise level of the environment and the traits’ sensitivity on the environment. Environmental noise acts on the birth rate and appears in Eq. (1) multiplied by the number of individuals, *N*_*S*_, i.e. it is linearly multiplicative, which will be crucial for the results presented below. Beside environmental noise, also demographic fluctuations arising from the stochastic nature of the birth-death dynamics are present. Such fluctuations are more pronounced for smaller populations since the impact of a random event on the average is larger then. They lead to a phenomenon called ‘random drift’ responsible for neutral evolution which causes extinction events even without selection as driving force. According to standard formulations, such demographic fluctuations here yield the term 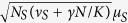, where 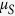 is *δ*-correlated noise, 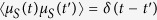, with a variance given by the sum of reaction rates^43^,^44^. We shall carry out further analysis for only two different traits 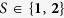 but generalizations to more traits are straightforward.

### Noise Correlation and Fokker-Planck Description

As it will turn out, the correlation level of the noise is crucial for some important features of the model. Environmental noise acting on the growth rate can influence several species at the same time or act independently on each species. To capture such different noise correlation levels we introduce a correlation parameter, 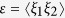. For instance, if several species feed from the same nutrients whose abundance fluctuates the noise of the growth rate of those species fluctuates with the same pattern (not necessarily the same amplitude). In this situation noise is perfectly correlated, i.e. 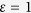. But also other situations are reasonable: If both species’ growth rates depend on different external variables, for example both feed from different carbon sources, noise is uncorrelated, 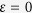. Also anticorrelated noise is potentially possible, *ε* = −1. This means that noise increases the growth rate of one species while it decreases the growth rate of another species. This might happen if one species metabolizes a substance which is poisonous for the other species. As all those scenarios are possible, we keep our analyses general by employing the correlation parameter, *ε*, and discuss scenarios where a particular choice of the noise correlation changes the evolutionary outcome.

To analyze the evolutionary dynamics and its dependence on both environmental and demographic noise, it is useful to study the Fokker-Planck equation (FPE) associated to Eq. (1). This equation cannot only be used to derive crucial quantities for the evolutionary process like fixation probabilities and times but also offers the possibility to distinguish the contributions of Darwinian fitness and neutral evolution as we are going to explain the the following section. In the remain of this section, the FPE for the relative abundance, 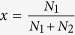, will be derived which might be skipped by a mathematically less interested reader. To do so, first the Langevin equations (1) have to be transformed to a FPE in the variables 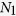 and 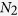. The procedure is straightforward but the correlation level of the noise has to be considered correctly^45^. This leads to the following FPE also depending on the correlation parameter *ε*,


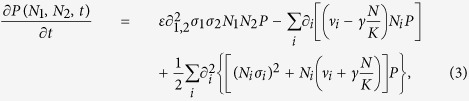


where 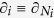. To uncover the influence of environmental noise on the evolutionary dynamics, the relative abundances seem the natural observables. Therefore, we change variables to the fraction 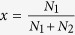 and the total number of individuals 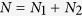. The FPE for *x* and *N* can be simplified exploiting the fact that the timescale of selection, 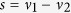, is much slower than the timescale for population growth 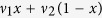. Therefore, we integrate over the total population size *N*, considering the FPE for the marginal distribution 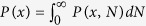, and employ 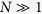, see Supporting Material (SM). The resulting one-dimensional FPE reads:


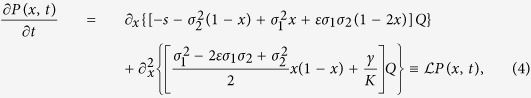


where 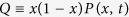 and the last equality defines the Fokker-Planck operator *L* needed in the following. For 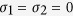, the drift term reduces to the well-know expression 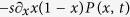 favoring the trait with the higher growth rate^46^. In ref. 15 a similar FPE was derived for the special case 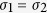. Note that, contrary to the analysis performed there, our model was first formulated considering the joint evolution of the total number of individuals and their relative abundances and then Eq. (4) for the relative abundances was derived by marginalization. That is crucial to fully capture the effects of noise.

### Sensitivity to Environmental Changes as an Evolutionary Disadvantage

With the one-dimensional FPE at hand, the evolutionary dynamics and especially the impact of environmental noise can be investigated. The equation has two terms: The first one proportional to 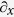 describes the drift due to selection on fitness differences and therefore corresponds to Darwinian evolution. Analyzing it gives us first insights about how the fitness depends on the sensitivity to environmental changes and which species is favored by selection. However, to fully understand the evolutionary outcome also the second term proportional to 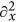 has to be considered. This term describes the impact of the random drift. For example if this term is much larger than the first one evolution is completely neutral and species outcompete other ones only by chance.

To grasp the consequences of different sensitivities to environmental changes, we first discuss the case of *distinct environmental sensitivities*, defined by 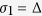 and 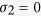, i.e. only the reproduction rate of the first trait depends on the environment. In Fig. 1B we show typical trajectories for such a scenario. There, we consider two species with the same average reproduction rate, 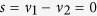 but different sensitivities 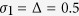 (green) and 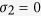 (violet). In the upper two panels the time evolution is shown when both species live in separate environments while in the lower panel their coexistence is considered. The question we are tackling in the following is whether or not there is an evolutionary force between those two species, in spite of their growth to the same total population size when living separated. The drift term in Eq. (4) which is proportional to 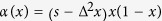 is key to answer that question. One can easily derive the mean field dynamics or deterministic limit from it,


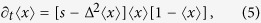


where 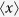 is the mean fraction of species **1** in the population. If *s* < 0, i.e. the second trait with a smaller variability in its birth rate is also faster in reproducing, the evolutionary dynamics does not change qualitatively compared to 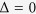. Conversely, if *s* > 0, the situation changes dramatically: the growth rate favors trait **1** while the variability term favors trait **2**. This leads to a stable fixed point 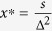 for 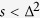 (for 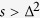 variability is not sufficient to prevent extinction of trait **2**). Such a dynamics can be interpreted as a frequency-dependent fitness function. However, the frequency-dependence arises here from environmental noise and not from a pay-off matrix as in standard evolutionary game theory^47^.

Even though environmental variability causes a drift term favoring the trait which is less sensitive to environmental changes^48^, the interplay between drift and diffusion term has to be understood to predict the evolutionary outcome. Indeed, the environmental contribution to the drift caused by 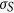 is intrinsically connected to the diffusion term. In other words whenever the fitness of a species is influenced by its sensitivity to environmental noise also neutral evolution is increased.Therefore we study the fixation probability, i.e. the probability that trait **1** fixates or trait **2** goes extinct. This quantity includes the effects of deterministic selection and random drift due to environmental and demographic noise and provides a complete picture of the evolutionary process. As detailedly shown in the SM, the fixation probability can be calculated by solving the backward FPE, 
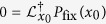
 associated with Eq. (4) for the boundary conditions 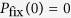 and 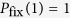 where *x*_0_ is the initial fraction of type **1**. The operator 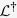 denotes the adjoint of *L* defined in Eq. (4). The solution of the backward FPE is given by,


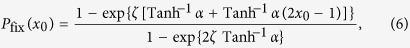


with 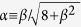 and 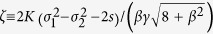 where 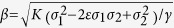.

**Figure 2:**
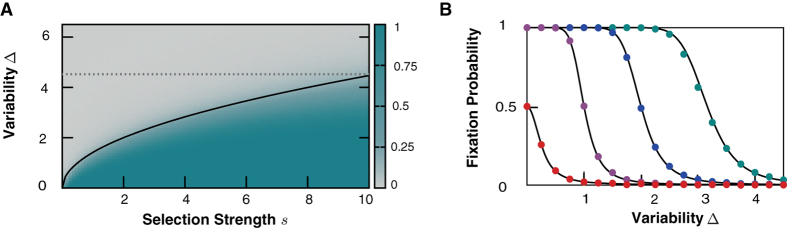
(**A**) Fixation probability, *P*_fix_, depending on selection strength, *s*, and variability 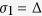 according to Eq. (1). In the green regime where 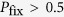 the faster growing trait is favored while in the gray regime the less sensitive species has an advantage. The black line indicates the parabola 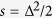, which is our prediction for 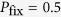. The dashed gray line corresponds to a condition for the survival of a non-competing species derived by May^18^. (**B**) Comparison of our analytic calculations for the fixation probabilities (Eq. (6)) and the full two species model (Eq. (1)) for 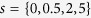 in {red, violet, blue, green}. Other parameters are 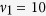, 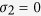, 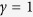 and *K* = 100 and *x*_0_ = 1.5.

In Fig. 2 we show the fixation probability for different values of *s* and 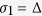 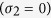 and compare it to our analytic calculations. Results are obtained by numerical solution of Eq. (1), i.e. before any approximation or simplification has been performed. One can clearly distinguish two distinct regions where one of the two species is is predominant: in the gray area the smaller variability dominates while in the green regime the faster growing species prevails. To test our hypothesis that the drift term and the resulting stable fixed point is responsible for this behavior we additionally plot the condition for a stable fixed point at 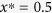 (solid black line) given by 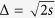. This line indicates the point in phase space where both species are equally likely to survive and therefore equally fit. As expected the line separates the two regimes where either the faster growing or the less sensitive species survives. In panel **B** of Fig. 2, the fixation probability depending on Δ is compared to the analytic solution (Eq. (6)) for four values of 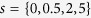. Both show very nice agreement proving that the approximations which were made to derive the one-dimensional FPE, Eq. (4), are valid and confirming conclusions drawn from it.

The general case of both species having variable birth rates yields analogous results: a selection advantage for the species with less sensitivity on the environment. Importantly, the selection disadvantage due to environmental noise does not scale with the carrying capacity as most demographic fluctuation effects do, i.e. the mechanism is effective irrespectively of the population size. When investigating only one species, the condition 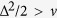 for a quick extinction of this species was derived in ref. 18 and is indicated in Fig. 2A by a dashed gray line. Our results manifestly go beyond that limit and a substantial disadvantage for the more variable species is present even when its variability is small enough to ensure survival in a scenario without competition.

An alternative interpretation of our results on the fixation probability is as follows. As mentioned above, the parameters *σ* or Δ depend on two factors: the sensitivity of a trait’s growth rate on environmental conditions and the strength of the environmental fluctuations themselves. For a given sensitivity of an individual on the environment, the ordinate Δ in Fig. 2 then corresponds to the strength of environmental fluctuations. While for weakly fluctuating environments a growth advantage is more beneficial, the situation is different for strong environmental variations. Then it is more advantageous to minimize the sensitivity to those variations rather than to optimize the growth rate. Interestingly one can construe this result in the context of game theory: decreasing the sensitivity to environmental changes also means to optimize the worst-case-scenario outcome because the average birth rate is the least reduced when the variability is small. In game theory, this corresponds to the MaxiMin strategy which was shown to be very successful in many fields as finance, economy or behavioral psychology^49^,^50^. In the field of evolutionary dynamics another example of a MaxiMin strategy is bacterial chemotaxis, where it was proposed that bacteria track chemoattractants trying to maximize their minimal uptake^51^.

### Neutral Evolution

Beside contributing to the fitness, environmental variability also influences fixation probability and time in the case of *neutral evolution*, i.e. 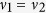 and 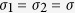. Such analysis is of great interest, as evolution is often studied by investigating how neutral mutations evolve over time. In recent years fast-sequencing techniques made huge amounts of data available^52^ which is now analyzed. Often it is interpreted by comparison to evolutionary models as for example the Moran or Wright-Fisher model^53^. In particular, the population size of ideal population which produces similar results in the toy model, the effective population size, is often inferred and used as characteristic quantity^54^. Interestingly it was observed that often thereby calculated population sizes are much smaller than the census population size. This observation is still present when one corrects for factors like a finite fertility span or the sex ratio^54^. In the following, we want to demonstrated that in addition to already know factors also environmental noise can account for a discrepancy between effective and census population size.

While the correlation parameter does not qualitatively influence results discussed so far, it plays an important role for neutral evolution. Interestingly, for fully correlated noise 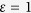, i.e. when both species are subjected to the same fluctuations meaning that even though the growth rates are variable their values are always the same, the resulting dynamics is exactly the same as in the case without environmental noise. Therefore, extinctions are solely driven by demographic fluctuations and well-known results apply^46^. Mathematically, this is reflected in Eq. (4), which for fully correlated noise, 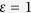, is the same as for 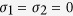. In contrast, for all other values of *ε*, including uncoupled noise *ε* = 0, the dynamics differs in two major respects. First, the drift term 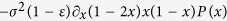 does not vanish and corresponds to a stable fixed point at 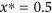 pushing the system to coexistence. Second, the diffusion term consists of demographic 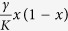 and environmental fluctuations 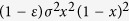 leading to a larger randomness in the evolutionary behavior. As the Darwinian drift suppresses extinction events while a larger diffusion term favors them, a more detailed analysis is required to grasp the evolutionary outcome.

**Figure 3:**
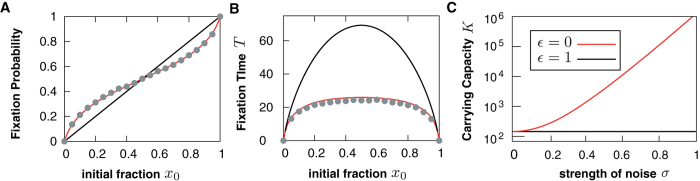
Fixation probability (panel **A**) and time (panel **B**) in the neutral case. Solid lines indicate analytical results for the two typical cases of perfectly correlated and uncorrelated noises *ε* = 1 and *ε* = 0. Parameters are: 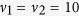, *K* = 100, 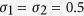 and *γ* = 1. Dots are simulations of the IBM and show good agreement with the analytic results. Additional parameters are 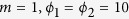, 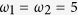, *τ* = 0.01, 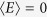, *α*_1_ = *α*_2_ = 1, and Var [*E*] = 100. (**C**) Reduction of the effective population size due to environmental noise in the neutral case for *x*_0_ = 0.5. Using (7), we plot the values of the carrying capacity *K* and noise *σ* that lead to the fixation time *T* = 100 for *γ* = 1. The black line corresponds to 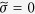 (see Eq. (7)), i.e. either perfectly correlated noise or no environmental noise. In the presence of environmental noise, the values of *K* are systematically higher and increase several orders of magnitude even for moderate noise levels.

Let us first consider the fixation probability [cf. Eq. (6)], see Fig. 3A. While for the standard situation of no environmental noise (or fully correlated noise) a linear dependence of the fixation probability on the initial fraction of a trait, *x*_0_ is observed, 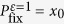, [Fig. 3A black line] for *ε* < 1 the situation is more complicated [red line]. Due to stable fixed point at 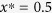, a S-shape arises. This means that the fixation probability for both species depends less on the initial fraction than in the standard case and both species are more equally likely to fixate.

While the behavior of the fixation probability is mainly due to the stable fixed point, the situation is more intricate for the extinction or fixation time which is another important quantity to describe evolutionary processes. As mentioned above, a coexistence fixed point is expected to increase the extinction time while a larger random drift decreases it. To ultimately understand the influence of environmental fluctuations, we therefore calculated the extinction time, 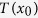, analytically. It also obeys a backward FPE, 
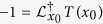
, which can be solved employing the boundary conditions 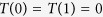, see SM:


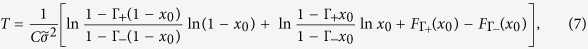


where 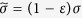, 
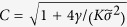
, 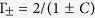, the function 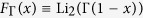

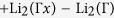
 and 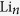 is the polylogarithm.

The result for not fully correlated noise (*ε* < 1) differs again from the non-fluctuating/fully correlated scenario, 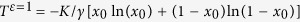 (see Fig. 3B). Fluctuating environments decrease the fixation times for all initial conditions despite the stable fixed point at 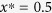. In other words, the extinction time is more strongly influenced by the larger neutral drift than by the stable fixed point. This has a crucial consequence: when measuring extinction times and comparing them to models without environmental fluctuations, one can only explain small fixation times which are due to larger fluctuations by demographic noise. Therefore, demographic noise has to account for all randomness and the resulting effective population size is much smaller than the census population size. Indeed, such a behavior is often found when analyzing data^54^. Figure 3C shows that conspicuous orders-of-magnitude reductions in the population size set in already at moderate levels of environmental noise, which could then account for differences between effective and census population size. In other words, as long as the strength of environmental noise and its correlation level are not known, the effective population size can only be interpreted as a lower bound for the census population size. In this context, environmental noise could also account for discrepancies between effective population sizes which are inferred with different methods for the same population. For instance environmental noise is not expected to change the population size determined via the Hardy-Weinberg equilibrium where the population size scales the sampling noise^54^ while it does when dynamic quantities like coalescence times are employed.

### Individual Based Model

To further investigate the impact of variable environmental conditions, we introduce an Individual Based Model (IBM). Such individual or agent based models serve as powerful tools to study evolutionary processes. They intrinsically include demographic noise as reproduction and death events are explicitly modeled. Additionally the IBM here serves as a proof of principle that linear multiplicative noise can be realistically expected when considering birth rates which depend on fluctuating environments. Since we model the environment and its fluctuations now explicitly, we can vary the environmental switching rate and thereby study the so far discussed phenomena beyond the white-noise limit, i.e. for environments which change slower. Even though, a particular choice for the IBM is made, we want to stress that the results presented above hold for any microscopic model whose macroscopic representation is given by Eq. (1), i.e. where the birth rate is subject to fluctuations thereby leading to linearly multiplicative noise.

In the IBM each individual reproduces according to its experienced environments. The simplest version of the model is that only the current environment influences the birth rate but to show that our results are more rigorous we also include more realistic scenarios where an individual’s environment history matters. To be more specific, the reproduction rate of an individual, *i*, at time *t*, depends a priori on the history of environmental conditions experienced during its lifetime 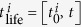, where 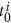 is the time of birth of the *i*-th individual. This could for example account for the accumulated level of nutrients or detrimental substances that individuals are exposed to. Following^55^,^56^, our model is based on independent birth and death rates, i.e. a birth event is not necessarily coinciding with a death event allowing for variably population sizes. The birth rates now depend on the environment which is described by a parameter *E*. Without loss of generality, we assume that larger values of *E* correspond to better environmental conditions. Depending on the correlation level of the noise, this parameter can be the same (*ε* = 1), completely independent (*ε* = 0) or correlated for different species. The number of environments experienced by an individual, *i*, is denoted as *M*^*i*^ and their values are contained in a vector 
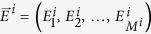
. Environmental conditions change stochastically at rate 1/*τ*. At each switching event a new environment is drawn according to a normal distribution, *p*(*E*), with mean 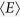 and variance 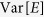.

**Figure 4:**
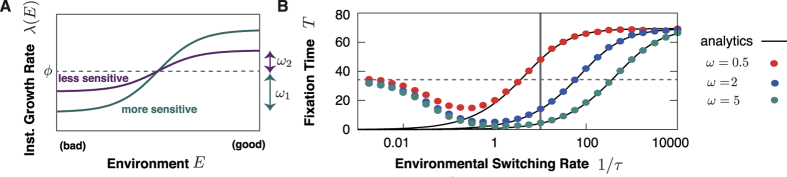
(**A**) Illustration of the instantaneous growth rate depending on the environment. Both species have the same average reproduction rate *ϕ*_1_ = *ϕ*_2_ = *ϕ* but species **1** is more sensitive to environmental changes (*ω*_1_ > *ω*_2_) (**B**) Comparison of the IBM and the Langevin model. We show the fixation time for neutral evolution for *x*_0_ = 0.5 *vs* the environmental switching rate 1/*τ*. Dots correspond to the IBM [*m* = 0] for different values of 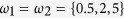 in red, blue and green. Black lines are analytic solutions [Eq. (7) with Eq. (10)]. For quickly fluctuating environments both results are in good agreement whilst for large *τ* the white noise approximation fails. The gray line corresponds to the timescale of reproduction. Other parameters are as in Fig. 3.

The growth rate of a species depends on the previously experienced environments. Before considering that, let us first discuss how the growth rate depends on a particular constant environment *E*. This quantity, the *instantaneous growth rate* 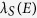 is assumed to be a increasing function of the environment *E* [see Fig. 4A], i.e. in better environments individuals reproduce faster. In particular, we consider the sigmoidal function:


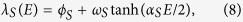


with *ϕ*_*S*_ the ordinate of the inflection point, 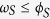 the maximal deviation from it, and *α*_*S*_ scaling the growth rate’s sensitivity to the environment.

Let us now consider changing environments and individuals whose current growth rate depends also on previously experienced environments. The reproduction rate 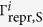 of an individual, *i*, of type *S* now depends on the whole vector, 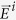. For concreteness, we assume that memory decays exponentially and that the rate is given by


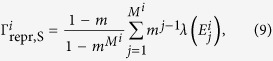


where the memory parameter 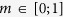 defines the influence of previously experienced environments upon an individual’s growth rate. For *m* = 0 only the current environment sets the growth rate 
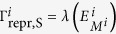
, and individuals do not memorize their past. In contrast, with increasing *m* the previously experienced environments become more and more important. For the limit 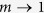 all experienced environments, *M*^*i*^, have the same influence and the growth rate is given by the arithmetic mean of all experienced instantaneous growth rates 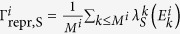. We assume for simplicity that offsprings lose memory at the time of reproduction, independently of *m*. As in the Langevin model, limited resources restrict the maximal number of individuals in the population. Therefore, the death rates 
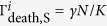
, increase with the total population size 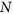.

#### Mapping

To compare the results of the microscopic individual based model to the effective stochastic model, Eq. (1), the parameters of both models have to be mapped. In this section, we briefly explain how such a mapping can be obtained but results in the following sections can be understood without those details. For simplicity let us consider the case 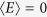 and a symmetric distribution *p*(*E*) throughout the following discussion. Since death rates are constant, there is a direct correspondence between their value in the Langevin and the IBM. For birth rates and their STDs the situation is more intricate as we discuss hereafter. For the no-memory case (*m* = 0) an exact mapping is obtained in the SM: For strong fluctuations, 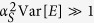, the mean of the growth rate 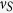 and the STD of the noise 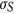 in Eq. (1) are given by:


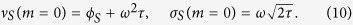


Note that the variability in the growth rate not only results in 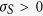, but also influences the average reproduction rate 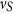. While for *m* = 0 such a variability increases 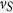, the second term of 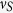 is reduced while *m* increases till it changes sign (see SM for details). For instance, for *m* = 1 the growth rate is approximately 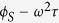. Hence, the more variable trait has a disadvantage in the average reproduction rate in addition to the effects discussed above. For *m* = 1, the approximation 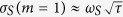 holds. Dependencies in this expression are intuited as follows. The number of environmental changes an individual experiences until the memory resets is of the order *M* ∼ *t*_life_/*τ*, where 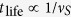 is the typical time for an individual to reproduce or die. As environmental changes are independent random events, the variance of the reproduction rates (9) is 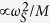. The expression for 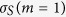 is finally obtained noting that correlations in the noise extend over times ~*t*_life_; it follows then that the average reproduction rate 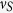 drops out.

#### Results Beyond the White-Noise Limit

For a detailed comparison of the IBM with the analytics derived in the first part of this paper, we simulate the IBM with a modified Gillespie algorithm updating reproduction rates after every environmental change^43^. As shown in Fig. 3A,B, results for fixation probability and time, are in excellent agreement with analytic solutions [Eqs. (6) and (7)]. In particular, the sigmoidal shape of the fixation probability is well reproduced by the IBM, supporting the existence and importance of linear multiplicative noise.

Finally, the IBM enables us to study the environmental switching rate. This is of main interest as previous results were obtained using a white-noise approximation and strictly hold only for very rapidly fluctuating environments. In Fig. 4B, the dependency on *τ* of the extinction time in the neutral case for 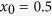 is shown for different *ω*_*S*_; see SM for results with 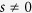. The black lines correspond to Eq. (7) mapped according to Eqs. (10) and dots are obtained by stochastic simulations of the IBM for *m* = 0. For *τ* < 1 both models are in very good agreement. For larger *τ* switching is too slow to be well described by white noise. For 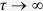 the environment never switches and as we choose a random distributed initial value of *E* the average fixation time is given by the mean average extinction time: 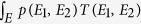 where 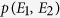 is the joint probability distribution that species **1** experiences environment *E*_1_ and species **2** experiences *E*_2_ and *T*(*E*_1_, *E*_2_) is the corresponding extinction time for constant environments. For large variances in the environmental distribution 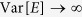 the expression can be further simplified. The environmental conditions then only fluctuate between a good and a bad state. Therefore, only a few different outcomes are possible: either both species are in the the good (respectively bad) state and the extinction process is neutral or they are in different states and extinction is selection driven. As the latter, is much quicker than neutral extinction it can be neglected in the mean. The resulting extinction time is now approximately given by the probability for neutral evolution, i.e. that both individuals experience the same environment, which is given by 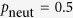 times the extinction time in the neutral case without environmental noise 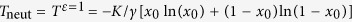. In Fig. 4B, the dashed line marks the value of this approximation.

All in all, our analysis of the IBM beyond the white noise limit confirms that there is a broad parameter regime where environmental fluctuations play a crucial role for both neutral evolution and the fitness functions. For fluctuations up to the timescale of reproduction events (marked by the vertical gray line), the description introduced above is valid. Nutrients and other metabolically important substances can vary on time scales quicker than reproduction. Therefore, we expect effects as discussed above to play a crucial role for evolutionary dynamics.

## Conclusion

We quantitatively demonstrated that environmental variability has crucial impact on evolutionary fitness. Our results do not rely on details of microscopic models but are rather derived from a macroscopic model whose only key assumption is that the birth rate of individuals is not constant but fluctuates. This assumption automatically leads to linearly multiplicative noise which gives rise to all discussed effects.

First, we quantified the role of reduced sensitivity to environmental changes and determined how it increases the fitness. Even though the increase stems from noise, its amplitude does not drop as the total population size increases. Therefore, such a mechanism is effective also for very large populations, contrary to most other fluctuation-based effects. By studying the interplay of the resulting evolutionary dynamics with random drift, we confirmed the importance of that fitness contribution and showed that those contributions are visible in a broad parameter regime. Importantly, even though fluctuation driven the fitness contribution due to environmental noise is of the same order of magnitude than the contribution due to different growth rates and present for all population sizes. This finding is of great interest when thinking about whether a generalist or a specialist is evolutionary favored^57^,^58^. We can quantify that depending on the level of environmental noise two regimes are present: For strongly fluctuating environments it strongly pays off to be less sensitive to such changes (to be generalist) while for little environmental fluctuations is more beneficial to reproduce as quick as possible (be a specialist).

In addition, we showed that the timescale of extinction in the neutral case is strongly affected by environmental noise. That provides a possible contribution to the reduction of effective population sizes, which are often found experimentally to be much smaller than the census population size. The reason is that environmental fluctuations increase the random drift that automatically results in smaller effective populations size, even if the source of the larger fluctuations is not demographic noise. Finally, we investigated individual based models that generate the linear multiplicative noise considered here. We thereby demonstrate that our description holds for fluctuation time scales up to the time scale of reproduction events.

As a future perspective, it will be of interest to study other forms of multiplicative noise in more detail, e.g. a noise in the death rate *γ* that would lead to a nonlinear dependency of the noise on the number of individuals. Also the interplay between the noise-induced frequency dependence discussed here and the one resulting from payoff matrixes in standard evolutionary game theory is worth further investigation. Finally, the question as to how fluctuation effects are influenced by reproduction rates that depend on time - a realistic model extension - remains open. With no environmental fluctuations, such rates would result in a smaller standard deviation of the expected time of reproduction and could potentially further increase the strength of the effects on fitness that we presented here.

## Additional Information

**How to cite this article**: Melbinger, A. and Vergassola, M. The Impact of Environmental Fluctuations on Evolutionary Fitness Functions. *Sci. Rep.* **5**, 15211; doi: 10.1038/srep15211 (2015).

## Supplementary Material

Supplementary Information
