# Supplementary material for: The Impact of Environmental Fluctuations on Evolutionary Fitness Functions
Source: Sci Rep. 2015 Oct 19;5:15211. doi: 10.1038/srep15211 (PMC4609966; doi:10.1038/srep15211)
Supplement: Supplementary Information [file srep15211-s1.pdf]

Supporting Information: The Impact of Environmental Fluctuations on Evolutionary Fitness Functions

Anna Melbinger and Massimo Vergassola

ABSTRACT

In this supporting information, we provide more details to the calculations sketched in the main text. In particular, we derive the one component version of the Fokker-Planck equation and expressions for fixation probability and time. In addition, we show additional data for the individual based model. We especially focus on the derivation and discussion of the mapping depending on the memory of environments previously experienced by an individual.

In this Supporting Material, we provide more details on the calculations leading to the one-dimensional Fokker-Planck equation (FPE), Eq. (3) main text. Moreover, we present calculations for the average fixation time and the extinction probability. We discuss the mapping of the individual based model (IBM) to the Langevin model. For the no-memory limit we present an analytic derivation of the mapping. For other parameter values we give heuristic arguments that are supported by additional data. Finally, we present results for non-neutral evolution investigating the regime in which the white noise approximation is an adequate description for the evolutionary process.

1 Derivation of the one-dimensional Fokker-Planck equation

In this Section, we provide details how the Langevin equations,

$$\begin{aligned} \dot{N}_1 &= N_1(\nu_1-\gamma\tfrac{N}{K})+\sqrt{N_1\left(\nu_1+\gamma\tfrac{N}{K}\right)}\mu_1+\sigma_1 N_1\xi_1 \\ \dot{N}_2 &= N_2(\nu_2-\gamma\tfrac{N}{K})+\sqrt{N_2(\nu_2+\gamma\tfrac{N}{K})}\mu_2+\sigma_2 N_2\xi_2 \end{aligned} \quad \text{(S1)}$$

can be transformed into the one-dimensional FPE presented in the main text. Both noise terms in Eq. (S1) are interpreted in the Ito sense[?] because they only should act as noise, i.e. enter the variances but do not affect the means, as it can be seen in the Fokker-Planck equation (S4) [or (3) main text].

From general results on stochastic processes (see[1]), it follows that the previous Langevin equation is associated to the following two-dimensional FPE :

$$\frac{\partial P(N_1,N_2,t)}{\partial t}=\varepsilon\partial^2_{1,2}\sigma_1\sigma_2N_1N_2P-\sum_i\partial_i\left[\left(\nu_i-\gamma\frac{N}{K}\right)N_iP\right] + \frac{1}{2}\sum_i\partial_i^2\left\{\left[(N_i\sigma_i)^2+N_i\left(\nu_i+\gamma\frac{N}{K}\right)\right]P\right\}, \quad \text{(S2)}$$

where $\partial_i \equiv \partial_{N_i}$. The drift part is directly stemming from the non-fluctuating parts of the Langevin equations $N_S(\nu_S-\gamma N/K)$. Diffusion depends on the correlation level of the noises experienced by the two species. In particular, we have introduced the correlation coefficient $\varepsilon \equiv \langle\xi_1\xi_2\rangle/\sqrt{\langle\xi_1^2\rangle\langle\xi_2^2\rangle}$. The case when the two noises are the same is given by $\varepsilon=1$, when they are independent is $\varepsilon=0$ and when they are anti-correlated is $\varepsilon=-1$.

To study the evolutionary dynamics associated to Eq. (S2), the relative abundances are the natural choice of variables. Therefore, we transform the absolute abundances N_1 and N_2 to $x=\frac{N_1}{N_1+N_2}$ and $N=N_1+N_2$. To perform the change of variables, not only $N_1=xN$ and $N_2=(1-x)N$ have to be replaced, also the differential operators and the probability distribution have to be transformed. Ensuring that the latter is still normalized after change of variables, the Jacobian has to be introduced, $P(N_1,N_2)\to\frac{1}{N}P(x,N)$. The derivatives are given by, $\partial_{N_1}\to\frac{1-x}{N}\partial_x+\partial_N$ and $\partial_{N_2}\to-\frac{x}{N}\partial_x+\partial_N$.

After the change of variables, the FPE for x and N can now be further simplified exploiting the fact that the time scale of selection, $s=\nu_1-\nu_2$, is much slower than the one of the population growth $\nu_1x+\nu_2(1-x)$.[2] Therefore, we marginalize the FPE with respect to the total population size N. Thereby, the integrals $\int_0^\infty dN$ of N-derivative terms such as $\partial_N\bullet$ or $N\partial_N^2\bullet=\partial_N(N\partial_N\bullet)-\partial_N\bullet$ vanish and the FPE simplifies to

$$\begin{aligned}\frac{\partial P(x,t)}{\partial t} &= \partial_x\left\{\left[-s-\sigma_2^2(1-x)+\sigma_1^2x+\varepsilon\sigma_1\sigma_2(1-2x)\right]Q\right\} \\ &+\partial_x\left(\frac{s}{N}Q\right) \\ &+\partial_x^2\left\{\left[\frac{\sigma_1^2-2\varepsilon\sigma_1\sigma_2+\sigma_2^2}{2}x(1-x)+\frac{\gamma}{2K}+\frac{\nu_1-sx}{2N}\right]Q\right\},\end{aligned} \quad \text{(S3)}$$

where $Q\equiv x(1-x)P(x,t)$. The drift term in the second line stemming from demographic fluctuations can be neglected as $N\gg1$ holds. To finally arrive at the one-dimensional FPE employed in the main text, we compute the steady state population size N^*. As the deterministic differential equation for N is given by

$$\dot{N}=N\left[x\nu_1+(1-x)\nu_2-\gamma\frac{N}{K}\right],$$

the fixed point for the populations size is $N^*=K/\gamma[\nu_1x+\nu_2(1-x)]$. Employing that relation and the aforementioned

condition $s \ll \nu_1 x + \nu_2(1-x)$, the last term in Eq. (S3) can be simplified as $\frac{\nu_1 - sx}{2N} \approx \frac{\gamma}{2K}$, which finally leads to the one-dimensional FPE in the main text:

$$\frac{\partial P(x,t)}{\partial t} = \partial_x \left\{ \left[-s - \sigma_2^2(1-x) + \sigma_1^2 x + \varepsilon\sigma_1\sigma_2(1-2x) \right] Q \right\} + \partial_x^2 \left\{ \left[\frac{\sigma_1^2 - 2\varepsilon\sigma_1\sigma_2 + \sigma_2^2}{2} x(1-x) + \frac{\gamma}{K} \right] Q \right\}, \tag{S4}$$

2 Fixation probability

In the following, we derive a general expression for the fixation probability. The calculations are analogous to the procedure for the neutral case described in the body of the paper. To determine the fixation probability the following backward equation has to be solved,

$$0 = x(1-x) \left\{ \left[s + \sigma_2^2(1-x) - \sigma_1^2 x - \varepsilon\sigma_1\sigma_2(1-2x) \right] \partial_x + \left[\frac{\sigma_1^2 - 2\varepsilon\sigma_1\sigma_2 + \sigma_2^2}{2} x(1-x) + \frac{\gamma}{K} \right] \partial_x^2 \right\} P_{\text{fix}}(x). \tag{S5}$$

Boundary conditions are $P_{\text{fix}}(0) = 0$ and $P_{\text{fix}}(1) = 1$. The solution to Eq. (S5) for the fixation probability is

$$P_{\text{fix}}(x) = \frac{1 - \exp\left\{ \zeta \left[\text{Tanh}^{-1}\alpha + \text{Tanh}^{-1}\alpha(2x-1) \right] \right\}}{1 - \exp\left\{ 2\zeta\, \text{Tanh}^{-1}\alpha \right\}}, \tag{S6}$$

with

$$\beta = \sqrt{K(\sigma_1^2 - 2\varepsilon\sigma_1\sigma_2 + \sigma_2^2)/\gamma}; \quad \alpha \equiv \frac{\beta}{\sqrt{8+\beta^2}}; \qquad \zeta \equiv \frac{2K\left(\sigma_1^2 - \sigma_2^2 - 2s\right)}{\beta\gamma\sqrt{8+\beta^2}}. \tag{S7}$$

The solution (S6) is obtained by integrating (S5) once, to find the gradient

$$\partial_x P_{\text{fix}}(x) = \text{const.} \frac{(1+\alpha(2x-1))^{\zeta/2-1}}{(1-\alpha(2x-1))^{\zeta/2+1}}. \tag{S8}$$

The expression (S8) is verified to be proportional to the derivative of $\left(\frac{1+\alpha(2x-1)}{1-\alpha(2x-1)}\right)^{\zeta/2}$ and boundary conditions are then imposed to fix the two constants of integration. The resulting expression is finally transformed into Eq. (S6) by using the elementary identity: $2\,\text{Tanh}^{-1}(x) = \log\left[(1+x)/(1-x)\right]$. It is verified that in the limit $\zeta \to 0$, one recovers the expression given in the main text.

All in all, the behavior we discussed in the main text is validated by analyzing the fixation probability: Both a higher growth rate and a smaller variability are beneficial for an individual.

3 Average time for fixation

3.1 Neutral case

The expression for the time of fixation in the neutral case that we presented in the body of the paper is derived as follows. The average time for fixation obeys the following backward equation,

$$\left\{ 1 - 2x + \left[x(1-x) + \frac{\gamma}{K\tilde{\sigma}^2} \right] \partial_x \right\} \partial_x T(x) = -\left(\tilde{\sigma}^2 x(1-x) \right)^{-1}, \tag{S9}$$

with $\tilde{\sigma} = (1-\varepsilon)\sigma$ the boundary conditions $T(0) = T(1) = 0$. Integrating Eq. (S9) and by variation of constants, we obtain:

$$\partial_x T(x) = \frac{1}{x(1-x) + \frac{\gamma}{K\tilde{\sigma}^2}} \left[A + \frac{1}{\tilde{\sigma}^2} \ln\left(\frac{1-x}{x} \right) \right], \tag{S10}$$

where A is a constant to be fixed by the boundary conditions. The integrals $\int_0^x$ of Eq. (S10) needed for $T(x)$ are performed by decomposing the rational function at the prefactor and using the formula :

$$\int \frac{\ln(a+bx)}{x} dx = \ln a \ln x - \text{Li}_2\left(-\frac{bx}{a} \right), \; a > 0, \tag{S11}$$

that follows from the very definition of the dilogarithm $\text{Li}_2(x) = -\int_0^x \ln(1-u)/u\, du$ (see[3]). The formula (S11) is used four times either directly (with a simple change of variables) or first integrating by parts to satisfy the condition $a > 0$ in (S11). The resulting expression is then transformed to the form given in the main text (which is the one given by Mathematica) by using the reflection property, $\text{Li}_2(x) + \text{Li}_2(1-x) = \text{Li}_2(1) - \ln x \ln(1-x)$, see.[3]

3.2 General case

In the general case when selection is present, the expression for the average fixation time cannot be found explicitly but is reducible to quadratures as follows. The fixation time obeys the backward equation (S5) with the left-hand side replaced by -1. Using the definitions (S7), we obtain

$$\left\{ 2\frac{K}{\gamma}(s + \sigma_2^2 - \varepsilon\sigma_1\sigma_2) - 2\beta^2 x + \left[\beta^2 x(1-x) + 2 \right] \partial_x \right\} \times \partial_x T(x) = -\frac{2K}{\gamma x(1-x)}. \tag{S12}$$

Boundary conditions are $T(0) = T(1) = 0$. The homogeneous solution was already found following (S8) and reads

$$T_{hom}(x) = C_1 + C_2 \left(\frac{\chi_+(x)}{\chi_-(x)} \right)^{\zeta/2}, \tag{S13}$$

where C_1 and C_2 are constants and we defined

$$\chi_+(x) \equiv 1 + \alpha(2x-1), \quad \chi_-(x) \equiv 1 - \alpha(2x-1), \tag{S14}$$

to simplify notation. The non-homogeneous solution for the gradient of T is obtained by varying the constant in (S8),

remarking that $\beta^2 x(1-x)+2 = \chi_+(x)\chi_-(x)(8+\beta^2)/4$ and integrating the resulting first-order differential equation to obtain

$$\partial_x T_{part}(x) = -\frac{K\chi_+(x)^{\zeta/2-1}}{2^{\zeta/2}\gamma(\zeta/2+1)} \times \left[(\alpha-1)F_1\left(\frac{\zeta}{2}+1,\frac{\zeta}{2},1;\frac{\zeta}{2}+2;\frac{1}{2}\chi_-(x),\frac{\chi_-(x)}{1+\alpha}\right)+ (\alpha+1)F_1\left(\frac{\zeta}{2}+1,\frac{\zeta}{2},1;\frac{\zeta}{2}+2;\frac{1}{2}\chi_-(x),\frac{\chi_-(x)}{1-\alpha}\right)\right]$$

where F_1 is the hypergeometric function of two variables.[4] The solution for T involves the integral $\int_0^x \partial_y T_{part}(y)\,dy$ of the expression above (for which a closed form does not seem to be available), and the two constants in (S13) are fixed by

$$C_1 + C_2\left(\frac{\chi_+(0)}{\chi_-(0)}\right)^{\zeta/2} = 0$$

$$C_1 + C_2\left(\frac{\chi_+(1)}{\chi_-(1)}\right)^{\zeta/2} = -\int_0^1 \partial_y T_{part}(y)\,dy\,.$$

It is verified from the expression above or directly from the original equation (S12) that in the two limits $x \to 0$ and $x \to 1$ the solution behaves like in the neutral case, *i.e.* $-K/\gamma x \log x$ and $-K/\gamma(1-x)\log(1-x)$. Selection and the rest of the parameters affect of course the solution in the rest of the interval of definition $x \in [0,1]$.

4 Coexistence time

Depending on the position of the stable fixed point, coexistence between two species (one with a larger growth rate, one with a smaller variability, $v_1 > v_2$ and $\sigma_1 = \Delta$, $\sigma_2 = 0$) is possible. In this section we present some additional data demonstrating this. In Fig. S1, the extinction time which corresponds to the time of coexistence is shown depending on Δ is shown for different values of s. Dots correspond to solutions of Eqs. (S1) and black lines are numerical solutions of Eq. (S12). The extinction time has a maximum which exactly coincides with the parameter values of a fixed point $x^* = 0.5$. The dependence of this maximal extinction time on the selection strength s is shown in Fig. S2.

5 Mapping individual-based models onto the Langevin dynamics

The aim of this Section is to show that individual-based models are described by the Langevin equations, Eqs. (S1), discussed in the main text and to analyze the mapping between the parameters of the two models.

The environmental conditions change stochastically at the rate $1/\tau$ and are distributed according to a distribution, $p(E)$, with mean $\langle E\rangle$ and variance Var[E]. The dependency of the instantaneous reproduction rate $\lambda_S(E)$ on E is given by the sigmoidal function :

$$\lambda_S(E) = \phi_S + \omega_S \tanh\left(\frac{\alpha_S E}{2}\right), \qquad \text{(S15)}$$

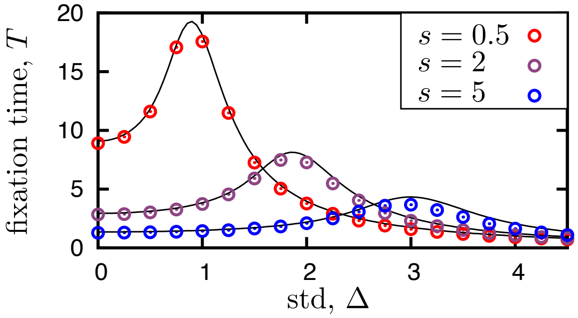

Figure S1. Extinction time depending on Δ for different values of the selection strength: $s = 0.5$ (red), $s = 2$ (violet) and $s = 5$ (blue). Dots are numerical solutions of the Langevin equations, Eq. (S1), and black lines are solutions of Eq. (S12).

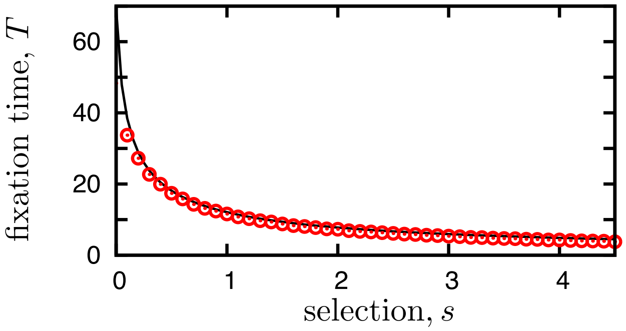

Figure S2. Extinction time for different values of s and $\Delta = \sqrt{(2s)}$. This combination of s and Δ corresponds to a stable fixed point at $x^* = 0.5$ and the maximal coexistence time for each value of s, see Fig. S1. As not only the selection strength but also the variability is increasing from left to right, the fixation time is a monotonically decreasing function of s.

which reduces to $\phi_S \pm \omega_S$ in the limit of large variances Var[E]. Birth rates are defined as,

$$\Gamma^i_{\text{repr,S}} = \frac{1-m}{1-m^{M^i}} \sum_{j=1}^{M^i} m^{j-1}\lambda(E^i_j). \qquad \text{(S16)}$$

In the no-memory limit $m = 0$, the growth rate is therefore given by the instantaneous growth rate $\lambda_S(E)$, while for $m \to 1$ the current growth rate is the arithmetic mean of all previously experienced environments. Death rates are given by $\Gamma^i_{\text{death,S}} = \gamma N/K$.

5.1 No memory, $m = 0$

We discuss first the model without memory, where the memory parameter, m, is zero : Individuals reproduce with the instantaneous reproduction rates [Eq. (S15)], which reduce to $\phi_S \pm \omega_S$ in the limit of large environmental variance. We consider an interval of length $\delta t \gg \tau$ such that the probability for

an individual to reproduce or die is small, yet the total number of events occurring over the whole population ($\sim K \gg 1$) is large. Neglecting the standard demographic noise term,[5] the variation of the S-type population is given by

$$N_S(t+\delta t) \simeq N_S(t) + N_S(t)\left(\phi_S - \gamma\frac{N(t)}{K}\right)\delta t + \\ + \omega_S \int_t^{t+\delta t} N_S(s)\hat{\sigma}(s)\,ds \tag{S17}$$

where $\hat{\sigma}(s)$ is the environmental Boolean random variable that takes values ± 1 and switches with characteristic time τ. The last term of Eq. (S17) is estimated as follows

$$\int_0^{\delta t} N_S(t+s)\hat{\sigma}(t+s)\,ds \simeq N_S(t)\mathscr{G}^e + \\ + N_S(s)\omega_S \int_0^{\delta t}\hat{\sigma}(t+s)\,ds \int_0^{s}\hat{\sigma}(t+s')\,ds' , \tag{S18}$$

where $\mathscr{G}^e$ is a Gaussian random variable having zero mean and variance

$$\mathrm{Var}[\mathscr{G}^e] = \int_0^{\delta t} ds \int_0^{\delta t} ds' \langle\hat{\sigma}(s)\hat{\sigma}(s')\rangle = 2\tau\delta t . \tag{S19}$$

Here, we used that $\langle\hat{\sigma}(t)\hat{\sigma}(t')\rangle = e^{-|t-t'|/\tau}$ and $\delta t \gg \tau$. The second term in Eq. (S18) is evaluated at the order δt using the same integral, Eq. (S19), and gives $N_S(t)\omega_S\tau\delta t$. Combining back all the terms, we conclude that the equation (S17) is equivalent to the Langevin equation (S1) with the mapping of the parameters

$$v_S = \phi_S + \omega_S^2\tau ; \quad \sigma_S^2 = 2\omega_S^2\tau . \tag{S20}$$

Note that the standard demographic noise term in Eq. (S1) should a priori include the fluctuating environmental term $N_S(t)\omega_S\mathscr{G}^e$ in the sum of the rates. In fact, it can be safely ignored as $\phi_S N_S \delta t \gg \omega_S N_S \sqrt{2\tau\delta t}$ due to $\phi_S \geq \omega_S$ and $\delta t \gg \tau$.

Finally, the factor 2 appearing in σ_S^2 in (S20) depends on the Poisson statistics of the environmental fluctuations. If the duration is fixed and equal to τ, Eq. (S19) becomes $\tau\delta t$. In that case, the corresponding mappings are $v_S = \phi_S + \omega_S^2\tau/2$ and $\sigma_S^2 = \omega_S^2\tau$. This is confirmed numerically in Fig. S3 where we show data for exponentially distributed (black) and fixed duration (red) environments. Solid lines are analytic solutions of the fixation time [Eq. (6) main text] employing the respective mappings.

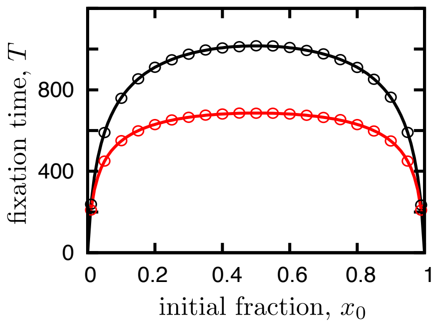

Figure S3. Comparison of data obtained by simulations in the neutral case with fixed (red) and exponentially distributed (black) environmental changes. Both sets of data agree with our analytic calculations, where we used the mappings $\sigma^2 = \omega^2\tau$ for fixed times of environmental changes and $\sigma^2 = \omega^2 2\tau$ for exponentially distributed switches. Thereby, the data confirms that the origin of the factor 2 in the mapping is solely the exponential distribution of the environmental changes. Parameters are $\phi_1 = \phi_2 = 1$ and $\omega_1 = \omega_2 = 0.9$, $\gamma = 1$, $K = 5000$, $\langle E\rangle = 0$, Var$[E] = 100$ and $\tau = 0.01$.

5.2 Finite memory, $m > 0$

We now turn to the scenario where memory extends over several environmental conditions that an individual previously experienced. Whilst for $m = 0$ an exact analytic mapping can be found in the limit of small τ, for finite memories the situation is more intricate. However, for the special case $m = 1$ the variability in the growth rate can be well approximated by the following argument. The reproduction rate $\Gamma^i_{\mathrm{repr,S}}$ of an individual, i, of type S at time t depends on the average of all instantaneous reproduction rates $\lambda_S(E)$ experienced by the individual :

$$\Gamma^i_{\mathrm{repr,S}}(t) = \frac{1}{M^i(t)} \sum_{k\leq M^i(t)} \lambda_S^k(E_k^i) = \phi_S + \tilde{\Gamma}_S^i(t) . \tag{S21}$$

At the time of reproduction, we assume for simplicity that offspring looses its memory of past environments experienced by the progenitor.

We consider again a time interval of length δt as in (S17). Fluctuations in the rates $\tilde{\Gamma}_S^i(t)$ decorrelate on timescales of the order of the lifetimes of individuals, t_{life}, which are much longer than τ and δt. Therefore on the δt scale, noise is smooth, contrary to (S17). Conversely, timescales of several lifetimes are much smaller than those on which selection acts and much longer than the characteristic time of the noise. Therefore, to describe the dynamics of the fractions, the environmental noises are well approximated by a shortly correlated noise. An estimation of the amplitude of the noise is obtained by calculating the sum

$$\sigma_S^2 \sim \frac{1}{\tau} \sum_{\ell=-\infty}^{\infty} \sum_{i=1}^{N_S} \sum_{j=1}^{N_S} \langle \tilde{\Gamma}_S^i(t_k)\Delta t_k \tilde{\Gamma}_S^j(t_\ell)\Delta t_\ell\rangle .$$

The durations Δt of the environmental intervals are independent for different k and ℓ (and the contribution $k = \ell$ is negligible with respect to the rest of the sum) so that one can replace them by τ. In addition, the symmetry in the indices of the intervals allows us to further simplify the expression

$$\sigma_S^2 \quad \sim \quad 2\tau \sum_{\ell=k}^{\infty} \sum_{i=1}^{N_S} \sum_{j=1}^{N_S} \langle \tilde{\Gamma}_S^i(t_k)\tilde{\Gamma}_S^j(t_\ell)\rangle . \tag{S22}$$

To compute the average in Eq. (S22) three different orders of events have to be distinguished: a) If the birth of the j-th individual was prior to the one of the i-th individual, then it follows from Eq (S21) that the quantity to be averaged is $\omega_S^2 M_k^i / \left(M_k^i M_\ell^j\right) = \omega_S^2 / M_\ell^j$, where M_ℓ^j is the number of environmental switches since the birth of the i-th individual up to time t_k. To derive Eq. (S22) we have used that the terms in the sum (S21) take independent values $\pm\omega_S$ with equal probability. Conversely, case (b) is when the birth of the j individual is posterior to the one of the i-th individual. Then the quantity to be averaged is $\omega_S^2 \left(M_k^i - \delta b\right) / \left(M_k^i M_\ell^j\right)$, where δb is the time between the birth of the i-th and the j-th individuals. Finally, in case (c) when $\delta b > M_k^i$, the correlation is zero as there is no overlap between the environmental fluctuations of the two individuals. Due to the Poissonian nature of the events, the number of switches since birth (back in the past) or before reproduction (forward in the future) have the same distribution $\exp(-M/\overline{M})/\overline{M}$ where $\overline{M} \sim t_{\text{life}}/\tau$ (its exact value does not affect the sequel). It follows that

$$\sigma_S^2 \sim \frac{2\omega_S^2 \tau}{\overline{M}^2} \left[\int_0^\infty dt \int_0^\infty du \int_0^\infty dv\, e^{-(t+2u+v)/\overline{M}} \frac{1}{u+v+t} + \int_0^\infty dt \int_0^\infty du \int_0^u dv\, e^{-(t+2u-v)/\overline{M}} \frac{u-v}{(u-v+t)\,u} \right]. \tag{S23}$$

The integral over t is the continuous approximation of the sum over $\ell - k$ appearing in Eq. (S22) while the variables u and v refer to the variables M_k^i and δb. The first and second term in the square parentheses of (S23) correspond to cases (a) and (b), respectively. By a series of change of variables and integrations by parts, it is shown that (S23) reduces to

$$\sigma_S^2 \sim \frac{2\omega_S^2 \tau}{\overline{M}^2} \int_0^\infty du\, e^{-u/\overline{M}} \int_0^u dv\, e^{-v/\overline{M}} = \omega_S^2 \tau\,. \tag{S24}$$

The validity of this approximation is confirmed for the neutral case in Fig. 2 in the main body of the paper where the fixation probability and time are compared to the analytic calculations employing Eq. (S24). Additional data for non-neutral evolution is presented in Fig. S4 where two species (one with finite variability, $\omega = 0.9$, one with vanishing variability) are analyzed. Analytic solutions for the fixation time and probability are fitted to simulation data. The best fit deviates less than 1% from Eq. (S24).

We now briefly discuss the dependence of σ_S on the memory parameter m. In Fig. S5, the STD of the noise in the growth rate, σ ,depending on the memory parameter, m, is analyzed. Results were obtained by simulating the neutral evolution case, measuring the fixation time and calculating σ employing the analytic expression for the extinction time [Eq. 6 main text]. For $m = 0$ the thereby obtained value agrees nicely with the mapping introduced above indicated by the red dashed line [Eq. (S20)]. With increasing memory, m, the STD of the noise, σ, decreases monotonically and approaches Eq. (S24) for $m \to 1$.

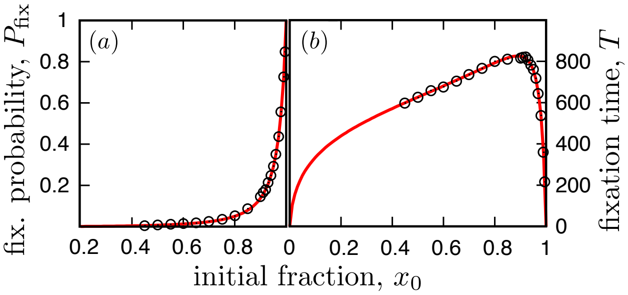

Figure S4. Fixation probability and time for non-neutral evolution with memory $m = 1$. Black dots correspond to simulation results of the IBM and red lines are analytic solutions. To obtain the latter we fitted Eq. (S6) and the solution of Eq. (S12) to the IBM. We used $\tilde{A} = s + \sigma_2^2 - \sigma_1\sigma_2\varepsilon$ and $\tilde{B} = \sigma_1^2 + 2\sigma_1\sigma_2\varepsilon + \sigma_2^2$ as fitting parameters and obtained $\tilde{A} = -3.04 \times 10^{-3}$ and $\tilde{B} = 8.25 \times 10^{-3}$. Other parameters are $\phi_1 = \phi_2 = 1$, $\omega_1 = 0.9$, $\omega_2 = 0$, $\tau = 1/100$, $\gamma = 1$, $K = 5000$, $\langle E \rangle = 0$ and Var$[E]$.

Let us now analyze the mapping of the average reproduction rate ν_S. Importantly, this mapping is very sensitive to model details which we will exemplify in the following. As results for neutral species do not dependent on the average reproduction rate, we have to turn to the evolution of non-equal individuals to understand the mapping of the growth rates. In Fig. S4, we show the fixation probability for two species with the same $\phi_1 = \phi_2 = 1$ but only the first species has a variable reproduction rate $\omega_1 = 0.9$, $\omega_2 = 0$ for $m = 1$. Red lines correspond to a fit with $s = \nu_1 - \nu_2 \approx -0.0030$ and agree perfectly with simulation results. In other words, the first species does not only have a disadvantage due its sensitivity on environmental changes, $\sigma_1 > \sigma_2$, but also has a smaller average growth rate. To study this effect in more detail, let us now analyze the fixation probability dependence on the memory parameter m, see Fig. S6 panel (a). Black dots correspond to the standard IBM (if not mentioned otherwise our discussion applies to this data), red dots to a slightly changed model which is going to be introduced in the following. For $m = 0$ both species are equally likely to fixate as the growth advantage of the more variable species $\nu_1 = \phi + \omega_1^2\tau > \nu_2 = \phi$ exactly compensates for its disadvantage due to the STD of the noise $\sigma_1 > \sigma_2$. For increasing values of m first the more variable ($m < 0.7$) later the less variable species is favored ($m > 0.7$). Whether this behavior is caused by the STD of the noise or differences in the mean reproduction rates is not obvious as the influence of both fitness contributions is of comparable strength. Therefore, we estimate the selection coefficient, $s = \nu_1 - \nu_2$, from the fixation probability data, see Fig. S6 panel (b). This is achieved by assuming that the variability of species **1** with $\omega_1 = 0$ is zero ($\sigma_1 = 0$) and that the variability of species **2** with $\omega_2 = 8.237$ is the same as in the neutral evolution scenario and thereby given by the data presented in Fig S5.

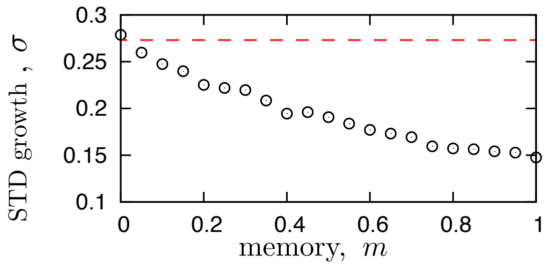

Figure S5. Numerical estimation of the STD of the noise dependence on the memory parameter m. Extinction times in the neutral scenario were measured. By evaluating the inverse of the fixation time function [Eq. (6) main text] the STD of the growth was calculated. For $m = 0$ the result agrees with the mapping, cf. Eq (S20), indicated by the red dashed line. For larger values of m the variability is reduced. Parameters are $\phi_1 = \phi_2 = 10$, $\omega_1 = \omega_2 = 8.237$, $\tau = 1/500$, $\gamma = 1$, $K = 100$, $\langle E \rangle = 0$ and $\mathrm{Var}[E]$.

Note that this approximation might neglect some higher noise correlations arising due to the coupling of both species via the carrying capacity. For $m = 0$ the thereby obtained value of s agrees well with our analytic results, cf. Eq. (S20). With increasing m the growth rate of the more variable species is decreased till the selection coefficients becomes negative effectively favoring the more variable species. However the decrease of the selection coefficient with m is smaller than the reduction of σ shown in Fig. S5. Hence, for small m the more variable species is favored as its advantage due to a larger average reproduction rate is larger than its disadvantage due to its sensitivity on the environment. This advantage in the growth rate is more sensitive to details in the IBM in comparison to the variability discussed in the main text for the Langevin equation Eq. (1).We are going to illustrate this by analyzing a slightly modified version of the IBM. But before doing so, we present an intuitive argument explaining one factor influencing the average growth rate: When an individual is born it experiences the current environment shorter than the average length of an environment. However, the model weights all experienced environments equally, see Eq. (7) in the main text. As the first experienced environment is more likely to be a good environment [more reproduction events happen during more beneficial environments], higher growth rates have a larger weight in the average and the average growth rate of the variable species is effectively increased. To obtain a description including this factor, it would be best to perform a time average over all previously experienced environments. Unfortunately, such a procedure is computationally very expensive. We therefore, test our explanation for the bias by not including the very first environment, the one in which an individual is born, in the averaged reproduction rate. The red dots in Fig. S6 corresponds to simulation results for this modified model. Even though all parameters are the same and for $\tau = 1/500$ and $\phi \approx 10$ an individual experiences in

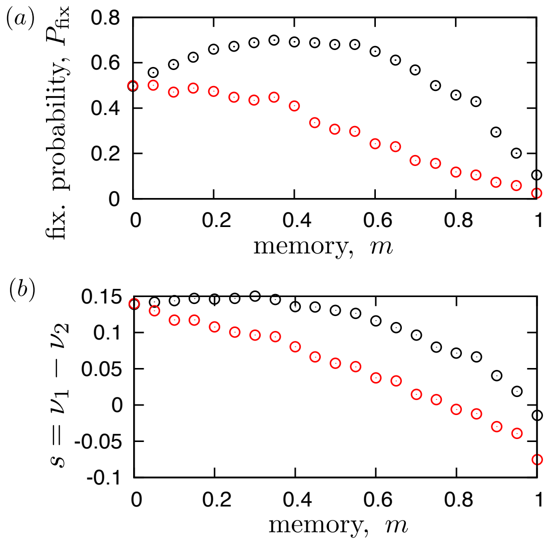

Figure S6. (a) Fixation probability and (b) selection coefficient depending on the memory parameter m. The first species' growth rate depends on the environment while the second one's is constant. Black dots correspond to the IBM introduced in the main body of the paper, while red dots represent a model modification not memorizing the first environment (the one in which an individual is born; for details see text). While the fixation probability is a direct simulation result, the selection coefficient s is inferred from it using the additional data presented in Fig. S5. Parameters are $\phi_1 = \phi_2 = 10$, $\omega_1 = 8.237$, $\omega_2 = 0$, $\tau = 1/500$, $\gamma = 1$, $K = 100$, $\langle E \rangle = 0$ and $\mathrm{Var}[E]$.

average 25 different environments, the small modification of the model substantially changes the simulation results. While the modification almost has no impact on the STD of the noise, it alters the average reproduction rate. For instance the regime in which the more variable species is favored completely disappears, cf. red dots Fig. S6 where $P_{\rm fix} \leq 0.5$ for all m. This example illustrates that on the first sight tiny details of an IBM might substantially influence the evolutionary outcome and that one should be cautious when drawing conclusions from them. Importantly, the mechanism discussed in the main text does not rely on specific assumptions of the microscopic models: A finite STD of the growth rate is always a disadvantage. It might be compensated for by a larger average reproduction rate but the same value of the growth rate without variability is always preferable.

6 DEPENDENCE ON THE SWITCHING RATE

In this Section, we present additional data for the non-neutral case. Fig. S7 shows the fixation probability depending on the environmental switching rate $1/\tau$. In particular, we investigate extinction for a species which is not sensitive to its environment ($\phi_1 = \phi = 10$, $\omega_1 = 0$) competing with a sen-

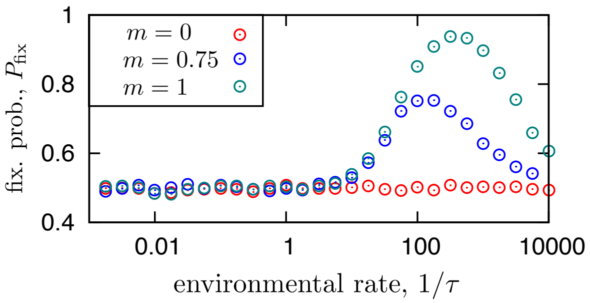

Figure S7. Dependence of the fixation probability on the environmental switching rate. Both species have the same $\phi_1 = \phi_2 = 10$, but only the second species is sensitive to the environment ($\omega_1 = 0,\ \omega_2 = 9$). For no memory ($m = 0$), both species are equally likely to fixate, as the advantage in the average growth rate of species **2** exactly compensates for its disadvantage due to its sensitivity on the environment. For $m > 0$ those two effects do not cancel out anymore and the second species is favored. Other parameters are $\gamma = 1$, $K = 100$, $\alpha = 1$, $\langle E \rangle = 0$ and $\mathrm{Var}[E] = 100$.

sitive species ($\phi_2 = \phi = 10$, $\omega_2 = 9$) for different values of m. In the case of no memory $m = 0$ both species are equally likely to fixate as the advantage in the average reproduction rate $\nu_2 = \phi + \omega_2 \tau$ exactly compensates for the disadvantage due to the STD of the noise in the growth rate [see Eq. (S20)]. For larger values of the memory parameter, a bias favoring the species with $\omega = 0$ is present (the exact value of the fixation probability depends on mapping details as discussed above). Importantly, the bias is not only present for very quickly fluctuating environments, but already emerges if reproduction events happen on a time scale comparable to τ. This supports the conclusion, that we were already drawing in the body of this paper when discussing Fig. 4: the white noise approximation is an adequate description for such an evolutionary process in that parameter regime.

References

1. Gnedenko, B. *Theory of Probabiility* (CRC Press, 1998).
2. Cremer, J., Melbinger, A. & Frey, E. Evolutionary and population dynamics: A coupled approach. *Phys. Rev. E* **84**, 051921 (2011).
3. Zagier. The dilogarithm function. In Cartier, P., Julia, B., Moussa, P. & Vanhove, P. (eds.) *Frontiers in Number Theory, Physics, and Geometry II: On Conformal Field Theories, Discrete Groups and Renormalization* (Springer-Verlag Berlin, 2007).
4. Gradshteyn, I., Ryzhik, I., Jeffrey, A. & Zwillinger, D. *Table of Integrals, Series, and Products* (Elsevier, 2007).
5. Kimura, M. *The Neutral Theory of Molecular Evolution* (Cambridge University Press, Cambridge, 1983).
